# Characterization of the human homozygous R182W *POLG2* mutation in mitochondrial DNA depletion syndrome

**DOI:** 10.1371/journal.pone.0203198

**Published:** 2018-08-29

**Authors:** Kirsten E. Hoff, Karen L. DeBalsi, Maria J. Sanchez-Quintero, Matthew J. Longley, Michio Hirano, Ali B. Naini, William C. Copeland

**Affiliations:** 1 Genome Integrity and Structural Biology Laboratory, National Institute of Environmental Health Sciences, National Institutes of Health, DHHS, Research Triangle Park, NC, United States of America; 2 Department of Neurology, H. Houston Merritt Neuromuscular Research Center, Columbia University Medical Center, New York, NY, United States of America; 3 Department of Pathology and Cell Biology, Columbia University, New York, NY, United States of America; 4 Division of Personalized Genomic Medicine, Department of Pathology and Cell Biology, Columbia University Medical Center, New York, NY, United States of America; University of Alabama at Birmingham, UNITED STATES

## Abstract

Mutations in mitochondrial DNA (mtDNA) have been linked to a variety of metabolic, neurological and muscular diseases which can present at any time throughout life. MtDNA is replicated by DNA polymerase gamma (Pol γ), twinkle helicase and mitochondrial single-stranded binding protein (mtSSB). The Pol γ holoenzyme is a heterotrimer consisting of the p140 catalytic subunit and a p55 homodimeric accessory subunit encoded by the nuclear genes *POLG* and *POLG2*, respectively. The accessory subunits enhance DNA binding and promote processive DNA synthesis of the holoenzyme. Mutations in either *POLG* or *POLG2* are linked to disease and adversely affect maintenance of the mitochondrial genome, resulting in depletion, deletions and/or point mutations in mtDNA. A homozygous mutation located at Chr17: 62492543G>A in *POLG2*, resulting in R182W substitution in p55, was previously identified to cause mtDNA depletion and fatal hepatic liver failure. Here we characterize this homozygous R182W p55 mutation using *in vivo* cultured cell models and *in vitro* biochemical assessments. Compared to control fibroblasts, homozygous R182W p55 primary dermal fibroblasts exhibit a two-fold slower doubling time, reduced mtDNA copy number and reduced levels of *POLG* and *POLG2* transcripts correlating with the reported disease state. Expression of R182W p55 in HEK293 cells impairs oxidative-phosphorylation. Biochemically, R182W p55 displays DNA binding and association with p140 similar to WT p55. R182W p55 mimics the ability of WT p55 to stimulate primer extension, support steady-state nucleotide incorporation, and suppress the exonuclease function of Pol γ *in vitro*. However, R182W p55 has severe defects in protein stability as determined by differential scanning fluorimetry and in stimulating function as determined by thermal inactivation. These data demonstrate that the Chr17: 62492543G>A mutation in *POLG2*, R182W p55, severely impairs stability of the accessory subunit and is the likely cause of the disease phenotype.

## Introduction

Mitochondria are cellular organelles in eukaryotic organisms responsible for producing ATP via electron transport and oxidative phosphorylation. Human mitochondria have their own genome comprised of a 16.5 kb circular DNA (mtDNA). Hundreds to thousands of copies of mtDNA exist within a cell depending on cell type and growth stage [1, 2]. The regions within mitochondria containing most of the mtDNA and associated proteins are referred to as nucleoids. These regions occur along the matrix side of the mitochondrial inner membrane. Super-resolution microscopy has shown recently that mitochondrial nucleoids contain 1–3 copies of mtDNA and that each cell can contain hundreds of nucleoids [3, 4]. MtDNA encodes 37 genes, including 13 components of the electron transport chain (ETC), 22 transfer RNA’s (tRNA’s), and 2 ribosomal RNA’s (rRNA). Mutation, deletion, and depletion of mtDNA are responsible for many mitochondrial disorders [5, 6]. Mitochondrial disorders range in age of onset from early infancy to late adulthood and present with a variety of overlapping phenotypes [7, 8]. Typically, early onset mitochondrial diseases result from mtDNA depletion, whereas late onset diseases result from mtDNA deletions [9].

Replication of mtDNA is performed by a core collection of nuclear-encoded proteins including: DNA polymerase γ (Pol γ), twinkle helicase and single-stranded DNA binding protein (mtSSB) [10]. Pol γ is the only known replicative mtDNA polymerase and functions as a heterotrimer comprised of one 140 kDa catalytic subunit (p140) and a 55 kDa homodimer accessory subunit (p55), encoded by nuclear genes *POLG* and *POLG2*, respectively. P55 has been characterized as a DNA binding protein that enhances and regulates p140 [11–18]. P55 functions as a processivity factor [11] and is unique among eukaryotic processivity factors due to its similarity to prokaryotic aminoacyl-tRNA synthetases [12, 19]. The symmetric p55 homodimer binds to p140 in an asymmetric manner, with each monomer playing a unique role. The p140-proximal subunit is reported to strengthen the DNA interaction while the distal subunit accelerates nucleotide incorporation [16, 17]. Although p55 is conserved among higher order eukaryotes, only vertebrate p55 is known to dimerize [20]. The affinity of p55 monomers to dimerize has been estimated at a K_d_ of ≤0.1 nM [21] and the apo structures reveal an extensive dimerization interface of about 4000 square angstroms [12, 19].

Mutations within the mtDNA replication machinery have been implicated in mitochondrial disease. Over 300 mutations have been identified within the *POLG* gene which are listed in the Human DNA Polymerase Gamma Mutation Database (https://tools.niehs.nih.gov/polg/). A small collection of heterozygous *POLG2* mutations has also been identified and analyzed both biochemically and *in vivo*. These include P205R, R369G, G451E and L475DfsX2, which were originally characterized as mutant homodimers [13, 22–24]. Recent work characterizing heterodimers with one WT and one mutant copy of p55 implicate the G451E as a strong dominant negative mutation [25]. *POLG2*^-/-^ knockout mice are embryonic lethal at day 8–8.5 demonstrating the importance of *POLG2* for survival, while heterozygous *POLG2*^+/-^ mice have a normal phenotype [26]. Null mutations of *POLG2* in *Drosophila melanogaster* lead to lethality in early pupal stage [27] and knockdown of *POLG2* in porcine oocytes stops oocyte maturation [28].

**Fig 1:**
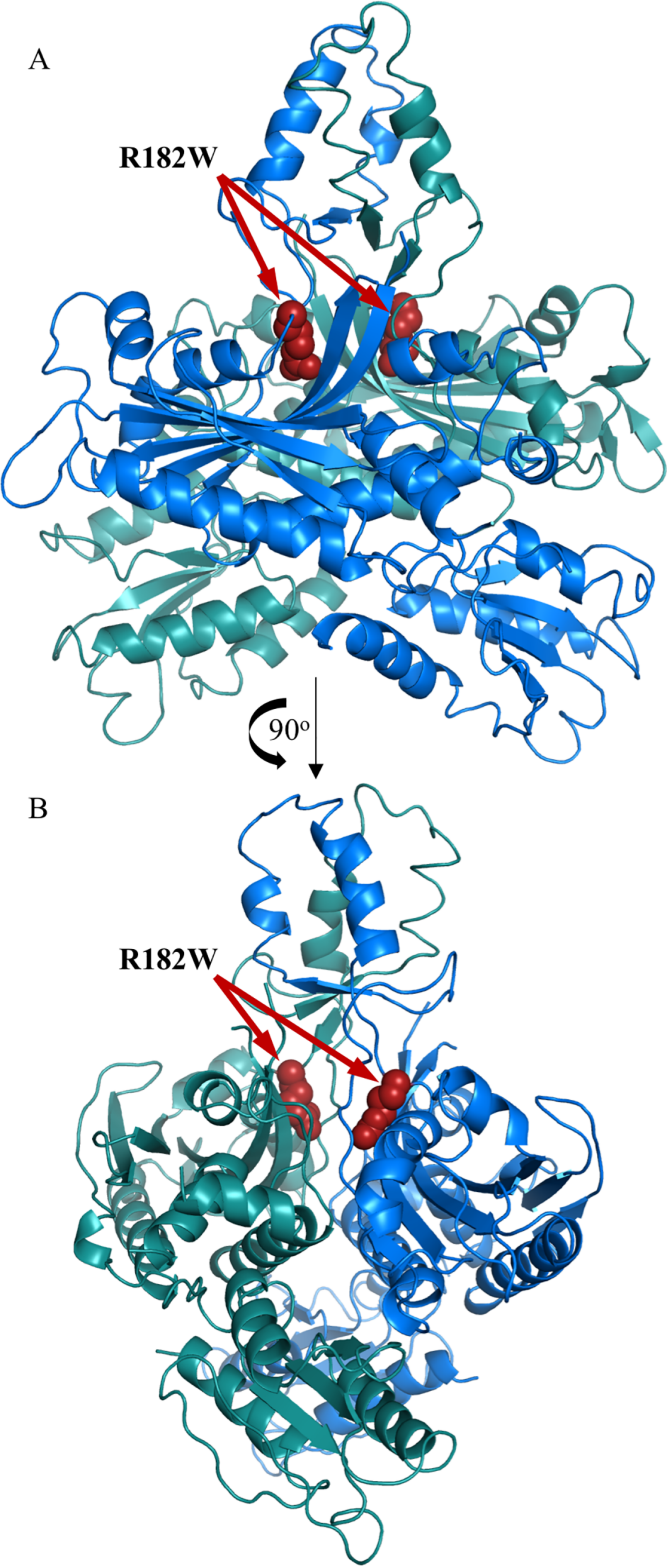
Location of the R182W amino acid substitution on the human apo p55 crystal structure. (A) Overview of the human apo p55 dimer structure. One monomer has been colored blue while the other monomer is colored green. Highlighted in red spheres is R182. (B) A 90^o^ rotation of the structure. PDB: 1G5H [12].

Recently, we identified the first homozygous *POLG2* mutation in a patient who presented with fulminant hepatic failure at three months of age and subsequently died at 9 months [29]. Severe mtDNA depletion was found in liver and muscle tissue along with partial depletion in blood lymphocytes. Whole exome sequencing identified a single homozygous point mutation located at Chr17: 62492543G>A which results in the R182W substitution. Both parents were found to be heterozygous for this mutation. To date neither parent has presented with mitochondrial disease. Structurally, this substitution is located at the dimerization domain base (Fig 1A & 1B). Mutation to a Trp causes loss of electrostatic interactions that may be important for stabilizing p55’s structure [29]. We hypothesized this mutation affects p55 dimerization, leading to loss of function. A publicly available phenotypic prediction algorithm, PolyPhen-2 [30], suggests that R182W is likely to ablate function (http://genetics.bwh.harvard.edu/pph2/). Interestingly, this residue is conserved among vertebrates with homodimeric p55 subunits, but not conserved among invertebrates, where only monomeric p55 has been observed [29].

To better understand the effects of R182W substitution in p55, we assessed growth rates, mtDNA copy number, and levels of select transcripts in patient dermal fibroblasts. We also studied the bioenergetics of HEK293 cells expressing R182W p55. Finally, we purified and characterized R182W p55 using a collection of biochemical assays, including comparative assessments of intrinsic affinity to dsDNA, stimulation of p140 polymerase and exonuclease steady state kinetics, enhancement of p140 processivity, physical association of p140 and p55, physical thermostability, and thermostable stimulation of p140.

## Materials and methods

### *POLG2* cloning

The R182W mutation was generated in the NHis-*POLG2* and pJ603-NGFP- *POLG2* [25] plasmids using the QuikChange site-directed mutagenesis kit (Stratagene) with the following mutagenic primers: Forward (5’—CT TCT GGG AAA CTA TGG GAG AAC CTT CTT CAC– 3’) and Reverse (5’—GTG AAG AAG GTT CTC CCA TAG TTT CCC AGA AG– 3’). Successful mutagenesis was confirmed by DNA sequencing the *POLG2* insert. The underlined nucleotides are changed by site-directed mutagenesis.

### Fibroblast cell culture

The research published here was approved by the IRB Office of Columbia University Medical Center (AAAB5754). With verbal informed consent from the parents, skin biopsies were obtained from the patient and were grown in 6-well plates using DMEM supplemented with 15% fetal bovine serum (FBS), 1% MEM vitamin solution and penicillin-streptomycin. With informed consent, skin biopsies were obtained from patients and were grown in 6-well plates using DMEM supplemented with 15% fetal bovine serum (FBS), 1% MEM vitamin solution and penicillin-streptomycin. Once they reached confluency, fibroblast cultures were expanded using 10% FBS DMEM in 10cm dishes. Neonatal dermal foreskin fibroblasts were purchased from ATCC (Catalog # PCS-201-010). Cells were cultured in either glucose or galactose containing DMEM. Glucose media consisted of low glucose DMEM (Catalog # 12320–032) supplemented with 100 U/mL penicillin, 100 μg/mL streptomycin, 0.25 μg/mL Gibco amphotericin B, 0.1 mM glycine, 0.1 mM L-alanine, 0.1 mM L-asparagine, 0.1 mM L-aspartic acid, 0.1 mM L-glutamic acid, 0.1 mM L-proline, 0.1 mM L-serine and 10% FBS. Galactose media consisted of DMEM without glucose (Catalog # 11966–025) supplemented with 10 mM HEPES, 100 U/mL penicillin, 100 μg/mL streptomycin, 0.25 μg/mL Gibco amphotericin B, 0.1 mM glycine, 0.1 mM L-alanine, 0.1 mM L-asparagine, 0.1 mM L-aspartic acid, 0.1 mM L-glutamic acid, 0.1 mM L-proline, 0.1 mM L-serine, 2 mM L-glutamine, 10 mM galactose, 1 mM sodium pyruvate and 10% FBS. Cells were grown at 37°C in a humidified atmosphere with 5% CO_2_ in air. Cells were routinely passaged by washing with PBS (pH 7.4), incubating with 0.25% trypsin, 1 mM EDTA for 2 min and then neutralized with media. Cells were next plated at 1:5 and 1:10 dilutions to maintain cells in the replicative growth phase. Vials of frozen cells were prepared in freezing medium containing 40% glucose DMEM, 50% FBS, 10% DMSO and stored in the gas-phase of a nitrogen freezer.

### Fibroblast growth rates

Growth curves of control and patient fibroblasts were performed in both glucose DMEM and galactose DMEM. Cells were passaged at least twice in the desired media before seeding for the growth curve. Control and patient fibroblasts were seeded in T-25 tissue culture flasks at 250,000 cells/flask at passage 7. Cells were grown at 37°C in a humidified atmosphere with 5% CO_2_ in air for 8 days. Three flasks were counted each day. To count the cells, they were washed with PBS (pH 7.4) followed by incubation in 1 mL of 0.25% Trypsin, 1 mM EDTA for 2 min and then neutralized with 1mL of the appropriate DMEM growth media. Cells were thoroughly resuspended, then counted in triplicate using a TC20^TM^ Automated Cell Counter (Bio-Rad). Total cell number was plotted versus time and the doubling times were calculated via *T_d_* = *T*×ln(2)/ln(*C_F_*−*C_I_*) where *T* is the time between the two cell counts, *C*_*F*_ is the final cell count and *C*_*I*_ is the initial cell count. The final and initial cell counts are based on the log phase of the growth curve which was determined to be between days 1 and 3.

### mtDNA copy number determination

1 x 10^7^ fibroblasts were harvested and total DNA isolated using the Qiagen AllPrep DNA/RNA/Protein mini kit following the manufacturer’s protocol. DNA was quantified by Qubit Fluorimetric quantitation (Thermofisher Scientific) using the dsDNA assay kit. RPPH1 was used as the control and both ND1 and ND4 regions were amplified to quantify mtDNA copy number. The target sequence for each gene was placed into the pCR2.1 vector for absolute quantification. 10 μL Real Time PCR reactions containing 5 μL 2x TaqMan Universal PCR master mix no AmpErase UNG (Applied Biosystems), 0.5 μL 20X TaqMan Primer–Probe set (*RPPH1* cat #: Hs03297761_s1, *ND1* cat #: Hs02596073_s1, *ND4* cat #: P14331348, Applied Biosystems), and 0.5 ng/μL genomic DNA were setup in a 384-well plate. Real time PCR amplification was performed using a QuantStudio 7 Real-Time PCR instrument (Applied Biosystems) with the cycling parameters being 95°C for 10 min, 95°C 15 sec, 60°C 1 min. The last two steps were performed for 40 cycles. Each of the three pCR2.1 vectors (RPPH1, ND1 and ND4) were used to create a standard curve with 10^2^, 10^3^, 10^4^, 10^5^, 10^6^ and 10^7^ copies/μL. Standard curves were repeated in triplicate for each gene on the plate. The DNA copy numbers were plotted against Ct values and linear regression was used to generate correlation coefficients. Copy number (*C*) for each gene was determined and then mtDNA copy number was calculated via *C* = *M/H* where *M* is the mtDNA genes (*ND1* or *ND4*) and *H* is the nuclear gene (*RPPH1*). Each sample was assayed five times on a single plate and experiments performed in triplicate.

### *POLG* and *POLG2* transcript level determination

1 X 10^7^ fibroblasts were harvested and total RNA isolated using the Qiagen AllPrep DNA/RNA/Protein mini kit following the manufacturer’s protocol. RNA was quantified by Nanodrop quantitation (Thermofisher Scientific). Complementary DNA (cDNA) was generated from 0.5 μg of RNA using the High-Capacity cDNA Reverse Transcription Kit (Thermofisher Scientific) following the manufacturer’s protocol. 10 μL Real Time PCR reactions containing 5 μL 2X TaqMan Universal PCR master mix no AmpErase UNG (Applied Biosystems), 0.5 μL 20X TaqMan Primer–Probe set (*ACTB* cat #: Hs1060665_g1, *POLG* cat #: Hs00160298_m1, *POLG2* cat #: Hs00945167_m1, Applied Biosystems), and 0.5 μL 1:2 diluted cDNA was setup in a 384-well plate. Real time PCR amplification was performed using a QuantStudio 7 Real-Time PCR instrument (Applied Biosystems) with the cycling parameters at 95°C for 10 min, 95°C 15 sec, 60°C 1 min. The last two steps were performed for 40 cycles. To calculate expression levels and fold change, the following equations were used: **Δ**Ct = Ct_GI_-Ct_Actin_, **ΔΔ**Ct = **Δ**Ct_P_- **Δ**Ct_C_, FC = 2^-**ΔΔ**Ct^ where Ct_GI_ is the average Ct value of either *POLG* or *POLG2*, Ct_Actin_ is the average Ct value for Actin, **Δ**Ct_P_ are the patient fibroblast values, **Δ**Ct_C_ are the control fibroblast values, and FC is fold change. Each sample was run five times on a single plate and experiments performed in triplicate.

### HEK293 cell culture and transfection

HEK293 cells (ATCC #CRL-1573) were cultured in Dulbecco’s Modified Eagle Medium (DMEM) (Life Technologies) without glucose (Catalog # 11966–025) and supplemented with 10 mM galactose, 6 mM L-glutamine, 10 mM HEPES, 1 mM sodium pyruvate, 100 U/mL penicillin, 100 μg/mL streptomycin, and 10% FBS. Cells were grown at 37°C in a humidified atmosphere with 10% CO_2_ in air. Cells were routinely passaged by washing with PBS (pH 7.4), incubating with 0.25% trypsin, 1 mM EDTA (Life Technologies) for 2 min and then neutralized with media. Cells were next plated at 1:10 and 1:20 dilutions to maintain cells in the replicative growth phase. Transfection was performed using the Lonza Nucleofector^TM^ 2b with the corresponding kit (Amaxa^TM^ cell line kit V) according to the manufacturers protocol. Cells were transfected with the previously described pJ603-NGFP-*POLG2* plasmid with and without the R182W mutation. Cells were transfected using the Lonza Nucleofector^TM^ 2b and then grown in the presence of 800 μg/mL G418 for selection. After 4–5 days, selection was maintained with 50 μg/mL G418. Vials of frozen cells were prepared in freezing medium containing 40% growth DMEM, 50% FBS, 10% DMSO and stored in the gas-phase of a nitrogen freezer.

### Cellular bioenergetics

Cellular bioenergetics were determined using the Seahorse Biosciences XF24 extracellular flux analyzer as previously described [25] with the following changes. Cells were cultured for 1–2 months in galactose media. HEK293 plates were seeded at 50,000 cells/well for untransfected and wild type p55 HEK293 cells and 70,000 cells/well for R182W p55 HEK293 cells and grown for two days at 37°C in a humidified atmosphere with 10% CO_2_ in air before assaying. Plates were checked for even seeding and 90–95% confluency. Data was normalized to cells seeded for analysis.

### P55 and p140 expression and purification

Wild-type (WT) and R182W p55 without the mitochondrial targeting sequence were expressed in BL21(DE3) *E*. *coli* as previously described [31] with one change. The nickel column elution buffer used to purify R182W p55 was modified to include 100 mM NaCl. All other buffers remained the same, ensuring that WT and R182W p55 are in the same buffer at the end of the purification. The exonuclease-deficient (Exo^-^) p140, herein referred to as WT p140, was overproduced in *Spodoptera frugiperda* (Sf9) insect cells and purified as previously described [31]. WT and R182W p55 proteins were assessed by SEC-MALS using an AKTA FPLC system (GE Healthcare) coupled to the miniDawn TREOS and Optilab rEX detectors (Wyatt Technology).

### Protein concentration determination

Bovine serum albumin (BSA) standards ranging from 0.5–7 ug along with the protein to be quantitated were run on 4–12% SDS-PAGE gels (Invitrogen) at 180 V for 60 min, then stained with Coomassie blue. Gels were imaged using a SYNGENE G:Box gel doc system and bands were quantitated with ImageJ64 [32]. The BSA standard areas were plotted against ug’s and linear regression used to generate correlation coefficients. The concentration of protein was then estimated against the BSA curve. BSA curves and the protein estimated were always run on the same gel.

### DNA binding electrophoretic mobility shift assay (EMSA)

For the apparent disassociation constants between DNA and WT p55 or R182W p55, the *K*_*d (DNA)*_ was determined by DNA Binding EMSA in triplicate as previously described [22].

### P140- p55 binding affinity

Holoenzymes were preassembled on ice in enzyme dilution buffer (EDB), composed of 50 mM Tris-HCl pH 7.5, 10% glycerol, 1 mM EDTA, 1 mM 2-ME, 100 mM KCl, and 0.005% NP-40, with a constant 2.5 nM p140 concentration and varying concentrations (0, 0.5, 1.0, 1.5, 2.0, 2.5, 3.0, 4.0, 7.5, 10.0, 15.0, 20.0, 25.0 nM) for WT and R182W p55. 50 μL reactions containing 25 mM HEPES-KOH (pH 7.5), 2.5 mM 2-ME, 0.5 mM MnCl_2_, 200 μg/mL heat-treated BSA, 16 μCi/mL [α-^32^P] dTTP, 50 μg/mL poly(rA)•oligo(dT)_12-18_, 220 mM NaCl, varying concentrations of dTTP (0, 0.5, 1, 1.5, 2, 2.5, 3, 5, 10, 15, 20, 25 μM) and 5 μL of holoenzyme were incubated at 37°C for 9 min. Reactions were stopped by the addition of 1 mg/mL BSA in 0.1 M NaPP_i_ (0.2 mL) and cold 10% TCA (1 mL). The mixture was filtered through Whatman GF/C filters, washed with 1N HCl, rinsed with 100% ethanol then dried. TCA-insoluble reactivity was determined by liquid scintillation counting. Maximal polymerase activity for WT and R182W p55 was set at 100% and apparent K_d(p55)_ values were derived by nonlinear regression analysis of DNA polymerase activity at each concentration of p55 dimer, as described [33].

### Steady-state kinetics

DNA polymerase activity on poly(rA)•oligo(dT)_12-18_ using preassembled holoenzyme containing either WT or R182W p55 was determined as follows: 50 μL reactions containing 25 mM HEPES-KOH (pH 7.5), 2.5 mM 2-ME, 0.5 mM MnCl_2_, 200 μg/mL heat-treated BSA, 16 μCi/mL [α-^32^P] dTTP, 50 μg/mL poly(rA)•oligo(dT)_12-18_, 220 mM NaCl, varying concentrations of dTTP (0, 0.5, 1, 1.5, 2, 2.5, 3, 5, 10, 15, 20, 25 μM) and a fixed concentration of holoenzyme (1 nM p140 + 2 nM p55) were incubated at 37°C for 0, 3, 6, 8 and 10 min. Reactions were stopped by the addition of 1 mg/mL BSA in 0.1 M NaPP_i_ (0.2 mL) and cold 10% TCA (1 mL). The samples were then filtered through Whatman GF/C filters, washed with 1 N HCl, rinsed with 100% ethanol and dried before TCA-insoluble radioactivity was determined by liquid scintillation counting. Steady-state kinetic values were determined as previously described [11].

### Exonuclease inhibition

Exonuclease activity was determined as previously described [34]. The final holoenzyme concentrations were: 0.3 nM p140 + 0.6 nM WT p55 and 0.4 nM p140 + 0.8 nM R182W p55.

### DNA processivity

Processivity of DNA synthesis was assessed as previously described [11] at 0 mM or 150 mM NaCl, without the pre-incubation step or the DNA trap. Samples were run on 12% denaturing PAGE gels.

### Differential scanning fluorimetry (DSF)

WT and R182W p55 were diluted to 1 mg/mL in EDB. SYPRO Orange (Molecular Probes) was diluted to 1X from a 40X stock in H_2_O. In a 96 well plate 20 μL of protein was mixed with 2.5 μL of SYPRO Orange. Using an ABI Prism 7900 Sequence Detection System (Applied Biosciences) the samples were heated from 25°C to 95°C at a ramp rate of 4% with detection set to the ROX reporter. The peak of the first derivative of the thermal melting curve defines the melting temperature (T_m_) for each protein. Data was analyzed using DMAN [35] and MATLAB’s ThermoQ application (MathWorks).

### Thermostability activity assay

WT and R182W p55 were diluted to 2 nM in EDB on ice and aliquoted into 10 μL samples. WT p55 was pre-incubated at 75°C for 0, 1.0, 3.0, 5.0, 7.0, 9.0, 12.0, 15.0, 25.0, 30.0, 45.0, 55.0, 65.0, 75.0, 90.0, 105.0, 120.0, 135.0, 150.0, 165.0, and 180.0 min while R182W p55 was pre-incubated at 75°C for 0, 0.5, 1.0, 1.5, 2.0, 2.5, 3.0, 3.5, 4.0, 4.5, 5.0, 6.0, 7.5, 10.0, 15.0, and 20.0 min. Each time point was placed on ice until the entire series was completed. Addition of 1 nM p140 while still on ice permitted assembly of holoenzymes. Reactions (50 μL) containing 25 mM HEPES-KOH (pH 7,5), 2.5 mM 2-ME, 0.5 mM MnCl_2_, 200 μg/mL heat-treated BSA, 220 mM NaCl, 300 μM dTTP, 16 μCi/mL [α-^32^P]-dTTP, 50 μg/mL poly(rA)•oligo(dT)_12-18_ primer-template and 5 μL heat-treated holoenzyme were incubated at 37°C for 10 min. Reactions were terminated and processed as described above. Activity observed for reactions without heat treatment was set to 100%, and remaining activity was plotted against time of 75°C pre-incubation. Data were fit to an equation for exponential decay (N_t_ = N_o_e^-**λ**t^), which permitted calculation of half-lives for inactivating p55 function (t_1/2_ = ln2/**λ**).

## Results

### Patient fibroblasts show delayed growth rates

**Fig 2:**
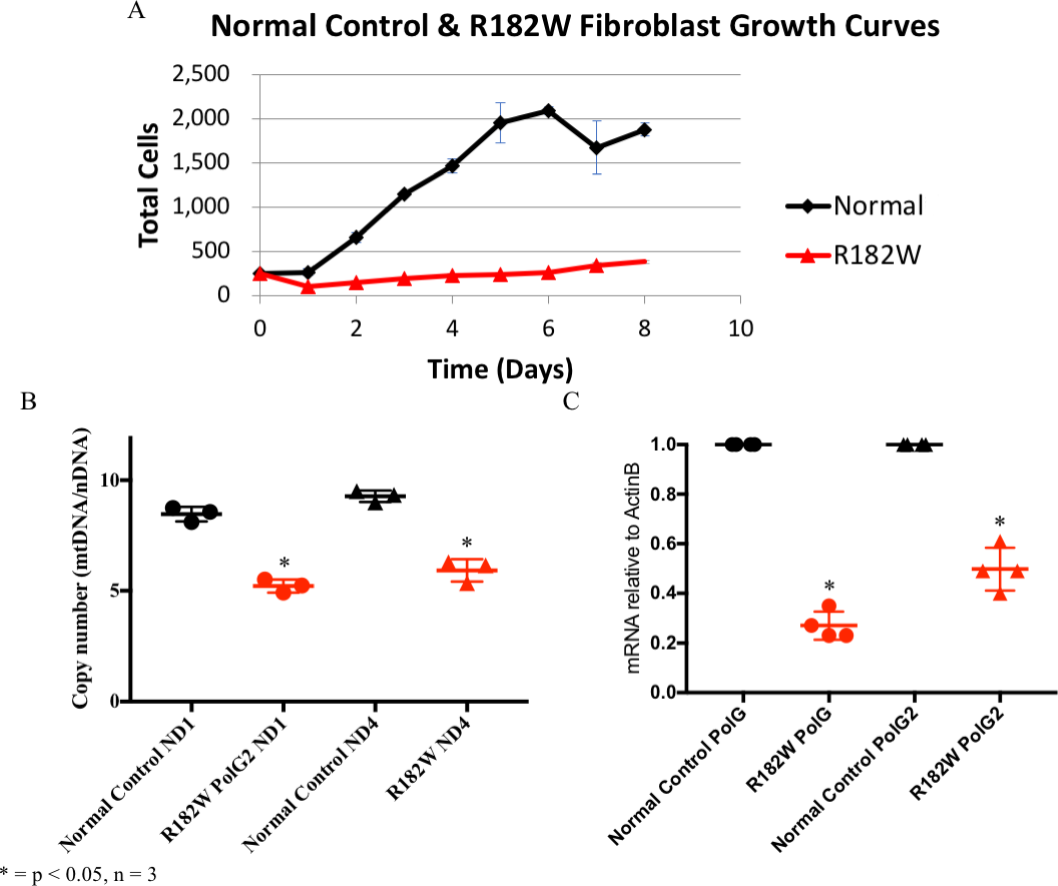
Patient dermal fibroblasts grow more slowly than control fibroblasts, have reduced mtDNA copy number and reduced *POLG/POLG2* transcript levels. (A) 8-day growth curves comparing control (black lines) and patient (red lines) dermal fibroblasts grown in glucose. Every point on the curves is the average of three independent growths. (B) MtDNA copy number was determined by quantitative PCR of the mitochondrial genes *ND1* and *ND4* and normalized to the nuclear gene *RPPH1*, as described in Materials and methods. MtDNA copy number for control fibroblasts (black circles–*ND1*/black triangles-ND4) or patient dermal fibroblasts (red circles-*ND1*/red triangles-*ND4*) are shown. Standard curves of plasmids containing the amplified regions of interest allowed for absolute copy number to be determined by real time PCR. Experiments were done in triplicate, and *N* = 5 for each data point. (C) Transcript levels for *POLG* and *POLG2* in patient fibroblasts were determined by quantitative PCR and normalized to *ACTB* as described in Materials and methods. WT p55 (black circles–*POLG*/black triangles–*POLG2*) R182W p55 (Red circles–*POLG*/red triangles–*POLG2*). Experiments were done in triplicate, and *N* = 5 for each data point.

To begin to understand the effects of the mutation at Chr17: 62492543G>A in *POLG2* (R182W p55) on cellular health and metabolism, growth rates of patient dermal fibroblasts were assessed in media with glucose or galactose. Requiring cultured cells to utilize galactose as a sole carbon source obligates the cell to rely on oxidative phosphorylation rather than glycolysis for energy production providing a simple test for severe respiratory chain defects [36–39]. When we switched the patient dermal fibroblasts to media containing galactose as the sole carbon source, we observed significant cell killing of the patient fibroblasts. Cells were seeded at 250,000 cells/T-25 flask at passage 7 and grown for 8 days with daily cell counting. Control fibroblasts had a doubling time of 22.7 hours in glucose while patient fibroblasts grew significantly slower with a doubling time of 56 hours in glucose (Fig 2A). Thus, the patient fibroblasts grew more than half as slow as the control fibroblast in glucose. In galactose, control fibroblasts had a doubling time of 24.5 hours while patient fibroblasts had a doubling time of 45.1 hours, suggesting patient fibroblasts were able to grow better in galactose than glucose. Although growth on galactose obligates mitochondrial respiration, the process of ensuring the fibroblasts have switched to aerobic respiration may be applying selective pressure and agrees with the observation of significant cell killing initially. Thus, only cells capable of surviving in those conditions grew and we likely artificially selected for healthier cells, making the galactose result uninformative.

### Patient fibroblasts have reduced mtDNA copy number

Since *POLG2* is essential for mtDNA replication, loss of function mutations in *POLG2* may cause a failure of mtDNA replication. The reduced growth rate for patient fibroblasts led us to determine mtDNA copy number to help establish whether R182W p55 is affecting mtDNA replication. Also, we had previously observed that the patient had reduced mtDNA copy number in liver and muscle (25 and 19% of wild type, respectively) [29]. MtDNA copy number was determined using absolute quantitative real time PCR. *ND1* and *ND4* amplification levels (mtDNA) were normalized to nuclear *RPPH1* levels to determine relative mtDNA copy number, and standard curves were used to calculate absolute mtDNA copy numbers. R182W p55 patient fibroblasts showed a significant decrease in mtDNA copy number with *ND1* being 61% of WT and *ND4* being 64% of WT (p ≤ 0.05, N = 3, as determined by Student’s T-test) (Fig 2B).

### Patient fibroblasts exhibit reduced p140 and p55 mRNA transcripts

Reduced mtDNA copy number could indicate either an inability of R182W p55 to stimulate processive DNA synthesis and/or a reduction in the level of p55 expressed within the cell. To help understand p55 function in the patient fibroblasts, we determined mRNA transcript levels of both p140 and p55 by quantitative real-time PCR. Transcript levels for both p140 and p55 were significantly reduced in glucose (20% and 40% of control cells, respectively, p ≤ 0.01, N = 3, as determined by Student’s T-test) (Fig 2C). This indicates that transcription of R182W p55 as well as p140 is reduced by the R182W mutation. The reduction of p140 and p55 transcripts may be due to coregulation of these genes [40] or, more likely, due to suppression of mitochondrial function in these patient fibroblasts when grown in glucose [39, 41]).

### HEK293 cells expressing R182W POLG2 have impaired respiratory capacity

**Fig 3:**
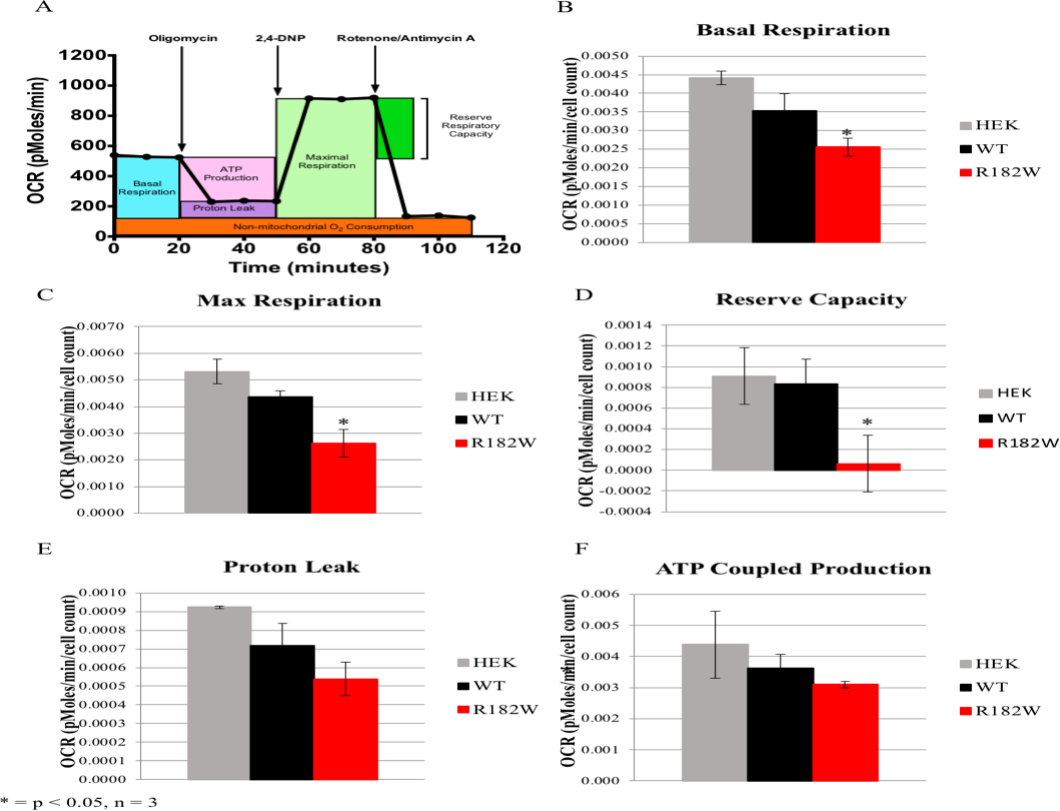
Seahorse Flux Analyzer bioenergetics analysis of transfected HEK293 cells illustrates significant reduction in respiration. (A) Representative graph of the oxygen consumption rate (OCR). ATP-linked oxygen consumption and oxygen consumption associated with proton leakage can be differentiated by inhibiting ATP synthase with oligomycin. Uncoupling electron transport and ATP synthase with 2,4-dinitrophenol (2,4-DNP) reveals the maximum rate of oxygen consumption. The reserve respiratory capacity is the difference between maximal and basal respiration rates. Complete inhibition of respiration using a combination of rotenone/antimycin A determines non-mitochondrial oxygen consumption. The average basal OCR (B), maximal OCR (C), reserve respiratory capacity (D), proton leakage (E) and ATP coupled production (F) for untransfected HEK293 (gray bars), WT POLG2 HEK293 (black bars) and R182W POLG2 HEK293 (red bars) cells are shown. Values are averages ± SEM, *N* = 3 per group, normalized to cells seeded. * *p* ≤ 0.05 versus the WT POLG2 transfected HEK293 cells as determined by Students t-test.

The patient fibroblasts showed an inability to produce mtDNA, presumably due to the R182W p55 mutation. However, these cells may have other unidentified mutations within either the nuclear or mitochondrial DNA. To confirm the observations in the patient fibroblast were caused by the R182W POLG2 mutation we episomally expressed either WT or R182W p55 in HEK293 cells. HEK293 cells were transfected with plasmids expressing WT p55 or R182W p55, and analysis of transcripts indicated a 5-fold overexpression of WT p55 compared to endogenous p55 in untransfected cells, and expression of R182W p55 was equivalent to endogenous p55 in untransfected cells. We have previously shown that overexpression of WT p55 does not alter bioenergetics in HEK293 cells [25]. We did not observe a difference in mtDNA copy number for R182W p55 or WT p55 HEK293 cells. We then sought to assess mitochondrial function using the Seahorse XF24 extracellular flux analyzer. The Seahorse XF24 extracellular flux analyzer utilizes a 24-well cell culture plate that analyzes pH and oxygen consumption of the cells to determine how well OX-PHOS is functioning. Inhibitors of oxidative phosphorylation are sequentially added that inhibit ATP synthase (oligomycin), uncouple Complexes I-IV from ATP synthase (2,4-dinitrophenol; 2,4-DNP), and then impede Complexes I/III (rotenone/antimycin A). The efficiency of OX-PHOS is determined by monitoring the oxygen consumption rate (OCR) during the addition of these compounds (Fig 3A). Despite equivalent levels of expression and mtDNA copy number, HEK293 cells expressing R182W POLG2 clearly show defective OX-PHOS with statistically significant decreases for basal respiration, maximal respiration, reserve capacity and ATP coupled production (* *p* ≤ 0.05 as determined by Students t-test, Fig 3B–3F).

### Recombinant expression and purification of R182W p55

The analysis of HEK293 cells expressing R182W p55 indicated that the R182W mutation can cause a defect in mitochondrial respiration. To better understand how the R182W p55 amino acid substitution affects biochemical function, the mutation was generated by site-directed mutagenesis, the recombinant protein was overproduced in *E*. *coli* and purified to homogeneity using the established protocol [11] with a single modification. The WT p55 is normally eluted from Ni-NTA with no additional salt added, but under these conditions, the R182W p55 precipitated during the elution. To ensure that the R182W p55 protein remained in solution the nickel elution buffer had to be adjusted to include 100 mM NaCl. This change can be made during WT p55 purification without affecting its function. The overall yield was 4-fold less than WT for equivalent starting material, which is sometimes indicative of structural/stability issues. Interestingly, analytical size exclusion chromatography indicated an apparent native molecular weight of 86 kDa for R182W p55, whereas WT p55 eluted as expected at 110 kDa. Otherwise, behavior throughout the purification protocol was similar to WT p55. To investigate whether the apparent native molecular weight was due to a shape change or an equilibrium between monomer and dimer, we performed size exclusion chromatography multi-angle light scattering (SEC-MALS). SEC-MALS data indicated that both R182W p55 and WT p55 exist as a single species (polydispersity of 1.000), suggesting a change in shape/conformation for the R182W p55 protein.

### R182W p55 DNA binding is similar to WT p55

P55 binds double strand DNA (dsDNA) independently of p140 [11], and dimerization of p55 is essential for dsDNA binding [42]. R182 is located at the base of the dimerization domain of p55 (Fig 1A & 1B). R182W amino acid substitution predicts a loss of electrostatic interactions between the monomers that could affect the structure of p55 and possibly alter dimerization [29]. Accordingly, we assessed DNA binding by R182W p55 as a functional screen for defects in dimerization. Various concentrations of WT and R182W p55 were incubated with a radioactively labelled 47-nucleotide double-stranded DNA oligomer as previously described [42]. Bound and unbound forms were resolved by electrophoretic mobility shift assay (EMSA), and apparent disassociation constants for WT and R182W p55 were shown to be indistinguishable (Table 1). This analysis suggests no defect in DNA binding by R182W p55.

**Table 1:** Biochemical characterization of p55.

	WT p55	R182W p55
**DNA binding**		
***K*_*d(DNA)*_** (nM)	89.6 ± 18.6	84.2 ± 22.3
**p140-p55 binding affinity**		
***K*_*d(p140)*_** (nM)*	0.35 ± 0.04	0.11 ± 0.01
**Steady-state kinetics**		
***K*_*m*_** (μM)	1.19 ± 0.1	1.08 ± 0.04
***k*_*cat*_** (min^-1^)	29.40 ± 0.91	34.65 ± 1.44
***k*_*cat*_/*K*_*m*_** (**μ**M•min)^-1^	27.64 ± 2.08	32.26 ± 1.39
**Modulation of p140-exonuclease activity by p55**		
Rate (nt/min)**	8.6 ± 2.3	14.0 ± 1.2

Table 1: DNA binding affinity, p140-p55 binding affinity, steady-state kinetics and exonuclease activity were determined as described in Materials and methods. Values are averages ± SEM, N ≥ 3.

*The p140-p55 binding affinity assay was performed at 220 mM NaCl.

**The exonuclease activity of p140 in the absence of p55 was 26.6 ± 0.8.

### Physical association of Pol γ is similar for R182W & WT p55

Known functions for p55 require association with dsDNA as well as association with the p140 catalytic subunit. Clinically identified mutations in *POLG2* have been shown to disrupt association of p55 and p140 [13, 22, 23]. We utilized a standard DNA synthesis assay with the homopolymeric primer-template substrate poly(rA)•oligo(dT)_12-18_ to assess the DNA polymerase activity of Pol γ holoenzyme under conditions of saturating dTTP and a restrictive salt concentration. At 220 mM NaCl, the DNA polymerase activity of p140 is insignificant without proper association with p55. Accordingly, p140 activity was assessed at varied and increasing concentrations of p55, and the formation of active holoenzyme is a measure of physical association of p140 and p55 subunits. The inter-subunit dissociation constant, *K*_*d(p140)*_ was calculated from a reciprocal plot of nucleotide incorporation as a function of p55 concentration. WT and R182W p55 were shown to bind p140 with similarly high affinities (0.35nM for the WT and 0.11nM for the R182W p55) (Table 1). While these numbers are statistically significant and suggest a slight increase in binding affinity for R182W p55, the biological significance for this modest tighter binding is unknown.

### Effects on steady-state polymerase kinetics by p55

At physiological salt concentration, it has been shown that p140 requires p55 to function [11, 43]. Steady-state kinetic parameters were determined for p140 in the presence of p55 in a standard DNA synthesis assay using the homopolymeric primer-template substrate poly(rA)•oligo(dT)_12-18_ with varying dTTP concentrations. Rates of dTMP incorporation over time were used to calculate apparent *K*_*m(dTTP)*_ and *k*_*cat*_ values with the Michaelis-Menten steady-state kinetic model (Table 1). No differences in the ability of R182W and WT p55 to modulate kinetic parameters were identified.

### P140’s exonuclease activity is influenced less by R182W p55 as compared to WT p55

P140 possesses a 3’-to-5’ exonuclease activity, which has an excision rate (*k*_*exo*_) of 26.6 ± 0.8 min^-1^ in the absence of p55 (Table 1 which is similar to previously published values [34]. It has previously been shown that p55 inhibits p140’s exonuclease activity by promoting processive DNA synthesis [15]. Here we examined the effect of WT and R182W p55 on p140’s exonuclease activity. Exonuclease activity was determined as previously reported [34] with the modification that p140 was preincubated with p55 in a 2:1 ratio. Final enzyme concentrations were determined experimentally to be within the linear range for this exonuclease assay and the steady state rate constant for excision of 3’-termini (*k*_*exo*_) was determined by plotting loss of substrate versus time over 5 minutes. In the presence of WT p55, p140’s *k*_*exo*_ was 8.6 ± 2.3 min^-1^ while in the presence of R182W p55, p140’s *k*_*exo*_ was 14.0 ± 1.2 min^-1^ (Table 1). This indicates that p55 impairs the exonuclease activity of p140 and that R182W p55 does not impair activity as dramatically as WT p55, suggesting a mild defect in association.

### Primer extension by p140 is similarly enhanced by R182W p55

**Fig 4:**
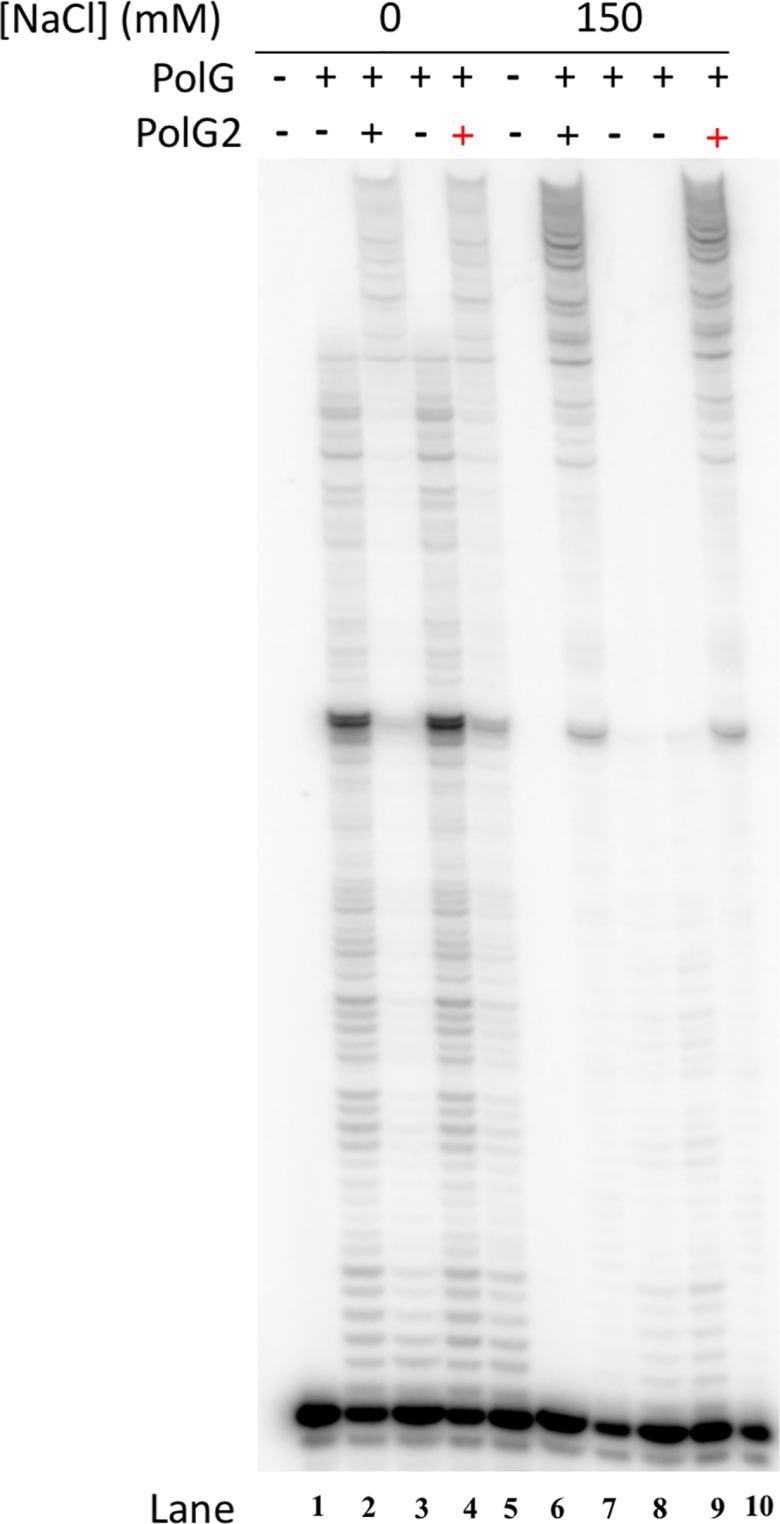
Stimulation of Pol γ processive DNA synthesis is similar for WT p55 and R182W p55. Primer extension reactions were performed and analyzed by denaturing PAGE as described in Materials and methods. Samples with or without p55 were assessed at 0 mM NaCl or at 150 mM NaCl, as indicated. Lanes 1 and 6 exclude enzyme. Lanes 2, 4, 8 and 9 exclude p55. Lanes 3 and 7 include WT p55 while lanes 5 and 10 include R182W p55. Lanes 1–5 have 0 mM NaCl while lanes 6–10 have 150 mM NaCl. The gel is representative of three independent experiments.

The p55 accessory subunit stimulates salt tolerant DNA synthesis by Pol γ and also enhances processive DNA synthesis by p140. On a primed M13 ssDNA primer-template, inclusion of p55 increases p140’s processivity from 50–100 nucleotides to several thousand nucleotides [11]. To compare further the effects of WT and R182W p55 on DNA synthesis by p140, we evaluated processivity of DNA synthesis in the presence and absence of each p55 form at both 0 mM and 150 mM NaCl concentrations using a gel-based primer extension assay that permits multiple DNA-binding events. R182W p55 enhances primer extension in a manner similar to WT p55 (Fig 4, compare lanes 3 and 5 or 7 and 10), indicating that R182W p55 functions to stimulate primer extension by p140 in a manner similar to WT p55.

### Thermostability of R182W p55 is significantly reduced

**Fig 5:**
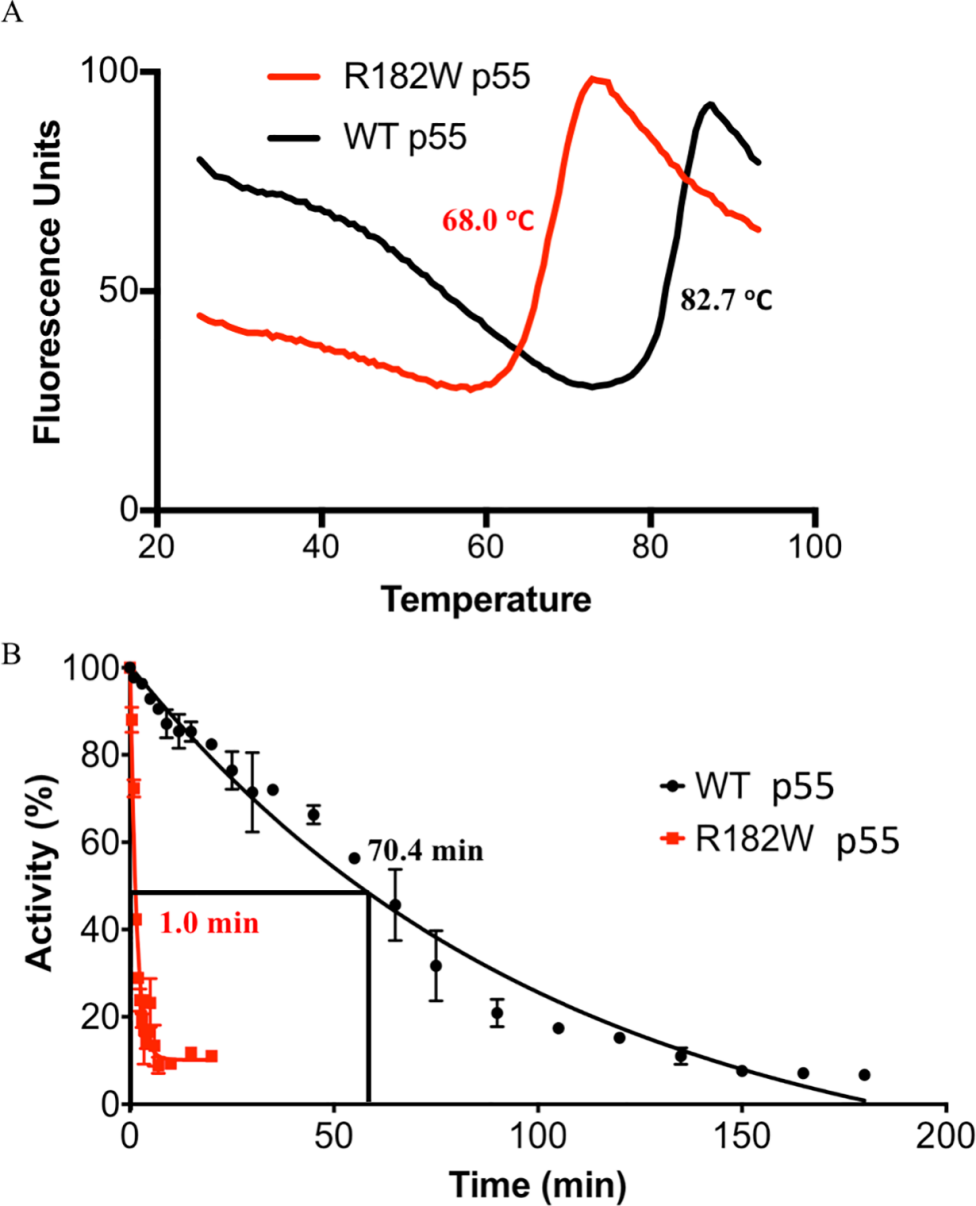
R182W p55 has a stability defect that disrupts p140 stimulation. (A) Differential Scanning Fluorimetry with SYPRO Orange dye. Protein was incubated with SYPRO Orange and warmed from 25°C to 95°C, as described in Materials and methods. Fluorescence of SYPRO Orange increases as more hydrophobic patches become available for binding, thereby reporting the degree of protein denaturation. R182W p55 has a lower melting temperature than WT p55. (B) Thermostability of WT p55 and R182W p55 was assessed in a p140 stimulation assay, as described in Materials and methods. WT p55 and R182W p55 were incubated at 75°C for increasing amounts of time, and holoenzymes were assembled and tested for the ability to incorporate dTTP. The half-life for thermal denaturation of R182W p55 is 70-fold shorter than the half-life for WT p55.

The purification procedure for R182W p55 required addition of 100 mM NaCl to the nickel column elution buffer to keep the protein in solution (see Materials and methods). This observation along with the SEC-MALS data suggested an altered stability for R182W-substituted p55. To assess protein stability, we utilized differential scanning fluorimetry to determine the melting temperatures for purified WT p55 and R182W p55. The fluorescent SYPRO Orange dye was mixed with each protein separately, and fluorescence of SYPRO Orange was monitored as the temperature was incrementally increased from 25°C to 95°C. As the protein unfolds, more hydrophobic residues become available to interact with SYPRO Orange causing an increase in fluorescence (Fig 5A). WT p55 exhibited a T_m_ of 82.7 ± 0.3°C while R182W p55 had a T_m_ of 68.0 ± 0.3°C (Fig 5A). This substantial decrease in melting temperature suggests a significant reduction in protein stability for R182W p55.

### Reduced thermostability of R182W p55 significantly impacts the ability to stimulate p140

With a reduced melting temperature, it became of interest to determine if exposure to increased temperatures would result in an inability to stimulate p140 in the standard DNA synthesis assay using the homopolymeric primer-template substrate poly(rA)•oligo(dT)_12-18_. In this assay, both WT and R182W p55 were heat inactivated at 75°C for varying lengths of time (WT up to 180 minutes, R182W up to 20 minutes). Pol γ holoenzymes were assembled *in vitro* with p140 and samples of each treated p55, and holoenzyme activity was evaluated by standard DNA synthesis assay. The half-life of WT p55 was determined to be 70.4 ± 4.4 min while R182W p55 was determined to be 1.0 ± 0.1 min (Fig 5B). This 70-fold reduction in the ability to stimulate p140 after heat treatment further indicates structural instability of R182W p55.

## Discussion

*POLG2* has been shown to be essential for life through a collection of studies that have revealed: (i) Homozygous knockout (*POLG2*^-/-^) mice die *in utero* at embryonic day E8.0–8.5, while heterozygous mice (*POLG2*^+/-^) are haplosufficient and develop normally [26]. (ii) Null mutations in *Drosophila melanogaster POLG2* cause lethality in the early pupal stage [27] and (iii) oocyte maturation is affected in a porcine *POLG2* knockdown model [28]. Until recently, the clinically identified mutations in *POLG2* were all heterozygous mutations [13, 22–24]. The Chr17: 62492543G>A *POLG2* mutation (R182W p55) was the first homozygous *POLG2* mutation identified, and the affected infant presented with liver failure at 3 months of age and subsequently died at 9 months [29]. We wanted to understand the effect of the R182W amino acid substitution on p55 function to validate the cause of the mtDNA depletion and understand the disease pathogenesis.

### *Ex vivo* studies

As an initial screen, we assessed the growth rates of patient dermal fibroblasts grown in either glucose or galactose media to understand whether the mutation at Chr17: 62492543G>A in *POLG2* (R182W p55) is detrimental to mitochondrial function. As expected, growth curves identified a growth defect for the patient fibroblasts. The doubling time was about twice as long as control cells. When we switched the fibroblasts to the galactose-based media significant cell killing was observed. Growth in galactose as the sole sugar source forces cells to produce energy via the OX-PHOS pathway thus ensuring we are examining mitochondrial energy production [36–38]. A subset of cells did survive, suggesting we ultimately selected for cells capable of surviving in the galactose media. Although dermal fibroblasts are easily obtained from patients; Robinson et al [39] noted approximately 50% of dermal fibroblasts from patients with mitochondrial disease are nonviable in galactose. Additionally, it has been reported that human epidermal cells are functionally anaerobic and thus do not require mitochondria for their viability [41]. Taken together, this suggests that fibroblast cells are not always the most appropriate cell type in which to study mitochondrial respiration. The most severely affected tissues in this patient were liver and muscle, however primary cells were not available for this study.

P55 functions to enhance p140’s replication of mtDNA, thus we determined the mtDNA copy number and mRNA transcript levels for *POLG* and *POLG2*. Both mtDNA copy number and transcript levels were significantly reduced when grown in glucose.

The patient fibroblasts grown in glucose confirm that mitochondrial dysfunction is present and suggest R182W p55 may be the cause. However, unidentified mutations may also be present within the fibroblasts. To isolate the effect R182W p55 has on cellular function, we expressed R182W p55 in HEK293 cells as previously reported [25]. These cells were grown in galactose media and cellular respiration was studied using the Seahorse XF24 extracellular flux analyzer. The R182W cells had significant defects in OX-PHOS function with the exception of proton leakage implying that R182W p55 may be detrimental to mitochondrial respiration. To determine the specific effect of R182W p55 substitution we purified and characterized R182W p55.

### Biochemical studies

The known biochemical functions of WT and R182W p55 were assessed *in vitro*. Double-stranded DNA binding affinity, p140 binding affinity, steady state stimulation of p140 DNA polymerase activity and stimulation of processive DNA synthesis by Pol γ were all similar for R182W p55 and WT p55. We found a reduction in the inhibition of exonuclease activity by R182W p55 as compared to WT p55. Together these results indicate that R182W p55 functions in a manner similar to WT p55 *in vitro*. However, during purification it was noted that the conditions had to be modified to obtain soluble protein. This suggested potential instability of the protein.

The R182W amino acid substitution lies at the base of the dimerization domain of p55 (Fig 1A & 1B), and this mutation disrupts a collection of electrostatic interactions potentially altering the structure of p55 homodimers [29]. Analysis by analytical sizing column indicates that R182W p55 has an apparent molecular weight of 86 kDa rather than WT p55’s 110 kDa, suggesting either a change in structure or an equilibrium between monomer and dimer exists. SEC- MALS data indicated that R182W p55 exists as a single species so an equilibrium is not present thus leaving a change in structure as the explanation. During purification of R182W p55, buffer modification was necessary to ensure R182W p55 would remain soluble. Taken together, these data suggest R182W p55 may have stability issues. To confirm this idea, we found that R182W p55 melts 14.7°C lower than WT p55 as determined by DSF. We further tested whether this difference in melting temperature would lead to an inability to stimulate p140. WT or R182W p55 were exposed to 75°C for a collection of time points, added to p140 and then assayed for polymerase activity under conditions requiring functional interaction of p55 with p140. This assay identified a large difference between WT and R182W p55, with the half-life for R182W p55 being 70-fold shorter than WT p55. Collectively, these results indicate that R182W p55 has a severe stability issue that results in loss of function. Recent studies indicate that oxidative phosphorylation derived respiration may result in an increased internal temperature of mitochondria of around 50°C rather than 37°C. One interesting possibility is that this higher temperature would exacerbate the stability defect of R182W p55 [44] and potentially lead to the presentation of disease in the clinic. Alternatively, unstable proteins provoke a variety of responses within living cells, including lowering of mRNA transcript levels, rapid degradation of expressed protein, blockages in the endoplasmic reticulum or the formation of amyloids [45, 46]. The patient dermal fibroblasts exhibit a dramatic reduction in transcript levels for both p140 and p55, supporting the idea that R182W p55 is unstable. Recently, lowered p55 protein levels as a result of a heterozygous splice site mutation in the *POLG2* gene was found responsible for adult onset syndromic sensory neuropathy, ataxia and parkinsonism and mtDNA deletions [47].

### Concluding remarks

We have characterized the first clinically identified homozygous mutation of human *POLG2*, R182W. Our studies indicate that when R182W p55 is folded properly and in solution, it functions similarly to WT p55. However, R182W p55 has a significant stability defect that dramatically impairs its ability to function. This stability defect is likely caused by the loss of electrostatic interactions normally afforded by the Arg side chain. *Ex vivo* studies indicate that patient dermal fibroblasts have a growth defect, reduced mtDNA copy number and lower transcript levels for both p140 and p55. HEK293 cells expressing R182W p55 have across the board reduced OX-PHOS capabilities. Taken together the results strongly suggest defects in the R182W p55 protein are responsible for mtDNA depletion in the patient and the subsequent onset of symptoms [29].
